# Pilot Study of Cytoprotective Mechanisms of Selenium Nanorods (SeNrs) under Ischemia-like Conditions on Cortical Astrocytes

**DOI:** 10.3390/ijms241512217

**Published:** 2023-07-30

**Authors:** Elena G. Varlamova, Egor Y. Plotnikov, Ilya V. Baimler, Sergey V. Gudkov, Egor A. Turovsky

**Affiliations:** 1 Institute of Cell Biophysics of the Russian Academy of Sciences, Federal Research Center “Pushchino Scientific Center for Biological Research of the Russian Academy of Sciences”, 142290 Pushchino, Russia; 2 A.N. Belozersky Institute of Physico-Chemical Biology, Lomonosov Moscow State University, 119992 Moscow, Russia; plotnikov@belozersky.msu.ru; 3 V.I. Kulakov National Medical Research Center of Obstetrics, Gynecology and Perinatology, 117997 Moscow, Russia; 4 Prokhorov General Physics Institute of the Russian Academy of Sciences, 38 Vavilovest., 119991 Moscow, Russia; ilyabaymler@yandex.ru (I.V.B.); s_makariy@rambler.ru (S.V.G.)

**Keywords:** selenium nanorods, astrocytes, apoptosis, necrosis, calcium ions, ischemia, reoxygenation, astrogliosis

## Abstract

The cytoprotective properties of the trace element selenium, its nanoparticles, and selenium nanocomplexes with active compounds are shown using a number of models. To date, some molecular mechanisms of the protective effect of spherical selenium nanoparticles under the action of ischemia/reoxygenation on brain cells have been studied. Among other things, the dependence of the effectiveness of the neuroprotective properties of nanoselenium on its diameter, pathways, and efficiency of penetration into astrocytes was established. In general, most research in the field of nanomedicine is focused on the preparation and study of spherical nanoparticles of various origins due to the ease of their preparation; in addition, spherical nanoparticles have a large specific surface area. However, obtaining and studying the mechanisms of action of nanoparticles of a new form are of great interest since nanorods, having all the positive properties of spherical nanoparticles, will also have a number of advantages. Using the laser ablation method, we managed to obtain and characterize selenium nanorods (SeNrs) with a length of 1 μm and a diameter of 100 nm. Using fluorescence microscopy and inhibitory analysis, we were able to show that selenium nanorods cause the generation of Ca^2+^ signals in cortical astrocytes in an acute experiment through the mobilization of Ca^2+^ ions from the thapsigargin-sensitive pool of the endoplasmic reticulum. Chronic use of SeNrs leads to a change in the expression pattern of genes encoding proteins that regulate cell fate and protect astrocytes from ischemia-like conditions and reoxygenation through the inhibition of a global increase in the concentration of cytosolic calcium ([Ca^2+^]_i_). An important component of the cytoprotective effect of SeNrs during ischemia/reoxygenation is the induction of reactive A2-type astrogliosis in astrocytes, leading to an increase in both baseline and ischemia/reoxygenation-induced phosphoinositide 3-kinase (PI3K) activity and suppression of necrosis and apoptosis. The key components of this cytoprotective action of SeNrs are the actin-dependent process of endocytosis of nanoparticles into cells and activation of the Ca^2+^ signaling system of astrocytes.

## 1. Introduction

The devastating effects of ischemic stroke affect millions of people each year, and current treatment options for this condition are severely limited. The development of better and safer treatments is hampered by a lack of knowledge about the precise pathogenic mechanisms underlying stroke. In 85% of stroke patients, the cause is an occlusion/blockage of an artery in the brain. Restoring blood flow (reperfusion) after a stroke remains a key strategy for stroke treatment [1,2]. However, two thirds of stroke survivors remain largely disabled. Although the timely restoration of blood flow in the cerebral artery is necessary to save brain tissue, reperfusion of the ischemic site, under certain conditions, also has devastating consequences, contributing to increased tissue damage with a further worsening of the disease outcome since it leads to oxidative stress [1,2]. Ischemic brain damage includes a cascade of signaling and metabolic events leading to the induction of necrotic and apoptotic processes. There is a decrease in the partial pressure of oxygen, the supply of tissue with glucose and other nutrients, an increase in the concentration of extracellular glutamate, an increase in [Ca^2+^]_i_ in neurons and astrocytes, an increase in ROS production, etc. [3,4].

The use of tissue plasminogen activator in the treatment of ischemic stroke has a number of limitations, the key ones being a narrow therapeutic window, lack of specificity, cell death, cerebral oedema, and damage to the blood–brain barrier (BBB) [5]. The delivery of drugs and imaging agents for the diagnosis of disorders of the nervous system is limited by the presence of two brain barriers—the blood–brain barrier (BBB) and the blood–cerebrospinal fluid barrier. Nanotechnology is currently extremely focused on obtaining nanostructures to overcome these barriers. The permeability of the BBB has been found to change in many CNS pathologies, such as brain injury, ischemic stroke, multiple sclerosis, Parkinson’s disease, Alzheimer’s disease, and psychiatric disorders such as mood disorders, psychosis, autism spectrum disorder, and even chronic sleep disorder [6]. There are two main ways of crossing the blood–brain barrier of the brain: paracellular and transcellular [7]. At the same time, it is believed that transcellular pathways transport molecules across the BBB more efficiently compared to the paracellular routes since they both use regular biological routes and retain the BBB integrity [8]. Nanoparticles are likely to be able to penetrate the BBB through both of these routes. Therefore, the search for effective nanocomplexes for the delivery of active compounds to brain damage centers remains an urgent task.

Nanoparticles are of great interest as a nanotransporter of active compounds to organs and tissues. Each of the forms of nanoparticles has its own advantages and limitations for use in biomedicine. Most works in the field of nanomedicine are focused on the preparation and study of spherical nanoparticles of various origins due to their ease of preparation and large specific surface area for sorbing active compounds [9,10,11]. However, there are prerequisites for the development of new, more complex forms of nanostructures to increase their therapeutic potential. It was shown that the relative bioavailability of the long rod loading drug system was much higher than that of short rod and spherical nanoparticles. However, there was no marked difference in the relative bioavailability of the short rod and spherical nanoparticles loading drug system. This result may be due to the following reasons. On the one hand, due to its longer residence time than spherical nanoparticles, the carriers possessed enough time to stay outside the intestinal mucosa and gradually released the drugs. On the other hand, long rods possessed longer blood-circulation lifetime after being absorbed by intestine than short rod and spherical nanoparticles. These two factors interacted together and make it have the highest relative bioavailability [9,12].

Selenium (Se) is extensively known as an important elemental semiconductor as well as an essential element in biology and chemistry. Se is a vital micronutrient for the body that acts through selenoproteins and selenium-containing glutathione peroxidases. Brain tissue is extremely sensitive to Se deficiency [13]. Se, acting through the activation of brain selenoproteins, is involved in the mechanisms of memory formation, and motor and cognitive activity [14]. Disturbances in selenoprotein expression and Se metabolism in the brain are associated with a number of neurodegenerative diseases, such as epilepsy, Alzheimer’s disease, Parkinson’s disease, etc. [14,15,16]. At the same time, it has been shown that exogenous Se and its compounds can alleviate the course of these diseases, primarily through suppression of oxidative stress, modulation of Ca^2+^ homeostasis, and mitochondrial biogenesis, i.e., contribute to maintaining the energy balance of brain cells and their survival in the penumbra zone [17,18]. There is strong evidence for the neuroprotective effect of selenium in the form of spherical nanoparticles [19,20,21]. Selenium nanoparticles (SeNPs) are metallic nanoparticles of elemental Se arranged as spherical particles, nanorods, nanowires, or nanotubes [22]. Spherical SeNPs as a source of Se for the body have been shown to be highly effective both as anticancer agents and as neuroprotectors [21,22,23,24,25,26].

The aim of this work was to comprehensively study the mechanisms of the cytoprotective effect of selenium in the form of a new nanoconstruct in the form of rods on cortical astrocytes in vitro at rest and during modeling of ischemia-like conditions (OGD) and reoxygenation.

## 2. Results

### 2.1. SeNrs Induce the Generation of Ca^2+^ Signals in Cortical Astrocytes

**Figure 1:**
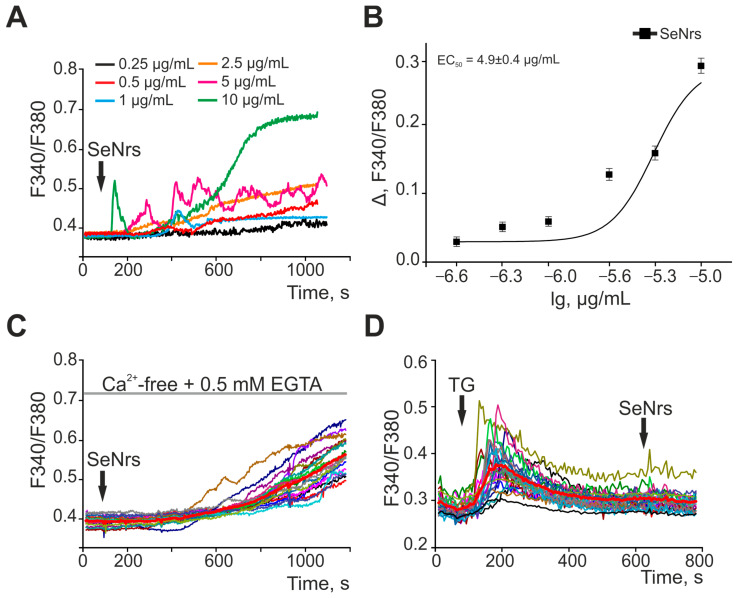
SeNrs dose-dependently cause the generation of Ca^2+^ signals in cortical astrocytes through the mobilization of Ca^2+^ ions from the thapsigargin-sensitive pool of the endoplasmic reticulum. (**A**) Ca^2+^ signals of astrocytes to the addition of various concentrations of SeNrs. Ca^2+^ responses of astrocytes to SeNrs averaged over several tens of cells are presented. (**B**) Dependence of the amplitude of Ca^2+^ responses of astrocytes on the growth of SeNrs concentration and its approximation by a sigmoid function. To plot dose dependencies, we used the results of Ca^2+^-dynamic measurements on three independent cell cultures. (**C**) Ca^2+^ signal generation by cortical astrocytes upon application of 5 μg/mL SeNrs in a calcium-free medium. (**D**) Suppression of Ca^2+^ signals of astrocytes upon application of 5 µg/mL SeNrs after depletion of Ca^2+^ stores in the endoplasmic reticulum with 10 µM thapsigargin (TG). F340/F380—The ratio of Fura-2 fluorescence in the 340 nm registration channel to the 380 nm fluorescence registration channel. Panels (**C**,**D**) show the Ca^2+^ signals of astrocytes in one experiment and their average value (bold red curve).

Previously, we found that nanoselenium in the form of spherical particles dose-dependently causes the generation of Ca^2+^ signals in astrocytes of various parts of the brain [27], and the EC50 for nanoparticles depended on their size [28]. In this study, application of SeNrs to cortical astrocytes led to the generation of Ca^2+^ responses starting from a concentration of 0.5 μg/mL (Figure 1A). Ca^2+^ signals of astrocytes are characterized by the generation of transient Ca^2+^ responses, followed by a gradual increase in [Ca^2+^]_i_ and the establishment of a new steady state [Ca^2+^]_i_ at concentrations of 5–10 µg/mL. Lower concentrations of SeNrs caused a gradual increase in [Ca^2+^]_i_ after a lag period of varying duration. An analysis of the dependence of the amplitude of the Ca^2+^ signal on the addition of SeNrs on the concentration of nanorods showed an increase in the level of [Ca^2+^]_i_ with an increase in the concentration of SeNrs and EC50 by 4.9 ± 0.4 μg/mL (Figure 1B).

To generate Ca^2+^ signals, cells can use both the entry of Ca^2+^ ions from outside and the mobilization of Ca^2+^ from intracellular pools. The addition of 5 µg/mL SeNrs in a nominally calcium-free medium in the presence of 0.5 mM EGTA caused the generation of Ca^2+^ signals in astrocytes (Figure 1C), with an amplitude comparable to the addition of SeNrs in the standard medium (Figure 1A). Emptying the Ca^2+^ pool of the endoplasmic reticulum (ER) by applying 10 μM thapsigargin (TG) prevented the generation of astrocyte Ca^2+^ signals in response to the addition of SeNrs (Figure 1D). Thus, SeNrs dose-dependently induced the generation of Ca^2+^ signals in the form of a gradual increase in [Ca^2+^]_i_ in cerebral cortex astrocytes due to the mobilization of Ca^2+^ ions from the thapsigargin-sensitive pool of the endoplasmic reticulum.

### 2.2. SeNrs Protect Cortical Astrocytes from OGD-Induced Death by Suppressing the Global Increase in Cytosolic Calcium Concentration

**Figure 2:**
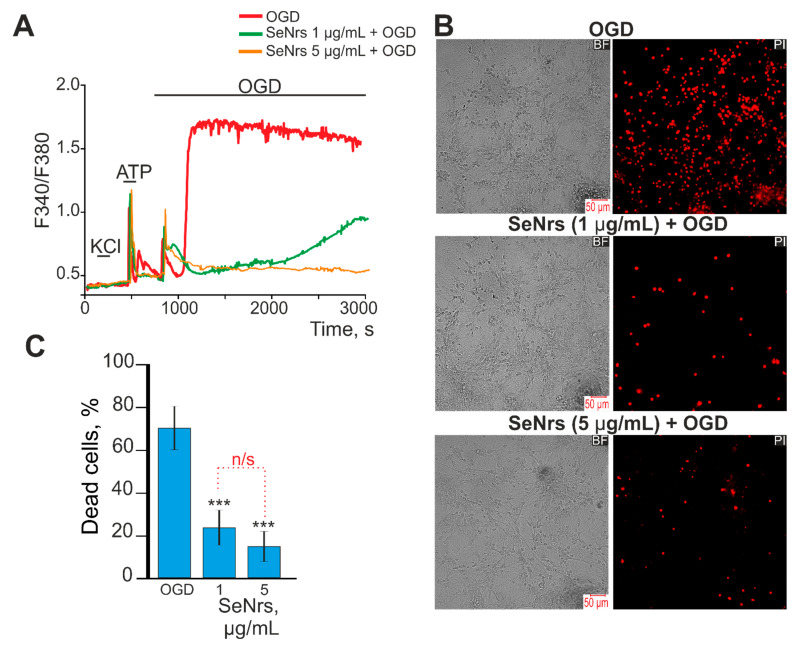
Effect of pre-incubation of cortical astrocytes with 1 or 5 μg/mL of SeNrs on Ca^2+^ signal generation and survival after OGD. (**A**) Ca^2+^ signals astrocytes during OGD (40 min) and OGD after 24-h pre-incubation with 1 or 5 μg/mL of SeNrs. The absence of Ca^2+^ signals on KCl indicates the absence of neurons in the cell culture. Ca^2+^ signals from astrocytes averaged over several dozens of cells are presented. The experiments were performed in three replicates on three separate cell cultures. F340/F380—The ratio of Fura-2 fluorescence in the 340 nm registration channel to the 380 nm fluorescence registration channel. (**B**) Images of cortical astrocytes loaded with propidium iodide (PI) after 40 min OGD. OGD conditions induced by omitting O_2_ in glucose-free media with argon bubbling. The red dots represent the PI-stained nuclei of necrotic cells. BF—bright-field microscopy. (**C**) Effect of 24-h pre-incubation with SeNrs on the cell viability after 40 min OGD. Black asterisks indicate the differences between the experimental groups compared with the OGD group. Differences between experimental groups are marked by red. *** *p* < 0.001, n/s—insignificant differences.

Preincubation of astrocytes with 1 or 5 μg/mL SeNrs for 24-h did not cause cell death (Supplementary Figure S1). Ischemia-like conditions (OGD) lasting 40 min induced a biphasic increase in [Ca^2+^]_i_ in astrocytes (Figure 2A, red curve). This correlated with the appearance of propidium iodide (PI) fluorescence after OGD (Figure 2B—OGD), which is a sign of necrotic cell death (Figure 2C). After a 24-h preincubation of astrocytes with 1 μg/mL SeNrs, there was a significant inhibition of the OGD-induced phase of the global increase in [Ca^2+^]_i_ (Figure 2A, green curve). In addition, the number of PI-stained cells decreased (Figure 2B—SeNrs (1 µg/mL + OGD), which may indicate suppression of necrosis (Figure 2C). Increasing the concentration of SeNrs to 5 μg/mL led to complete inhibition of the OGD-induced phase of the global increase in [Ca^2+^]_i_ (Figure 2A—orange curve) and suppression of necrosis (Figure 2B,C).

Thus, preincubation of astrocytes with SeNrs did not inhibit the first reversible phase of the OGD-induced [Ca^2+^]_i_ increase, but suppressed the phase of the global [Ca^2+^]_i_ increase, when 5 μg/mL SeNrs completely suppressed these astrocyte Ca^2+^ signals. Inhibition of the global increase in [Ca^2+^]_i_ led to a decrease in the number of astrocytes dying due to necrosis.

### 2.3. The Anti-Apoptotic and Anti-Inflammatory Effects of SeNrs during Ischemia/Reoxygenation Correlate with Reactive Astrogliosis

**Figure 3:**
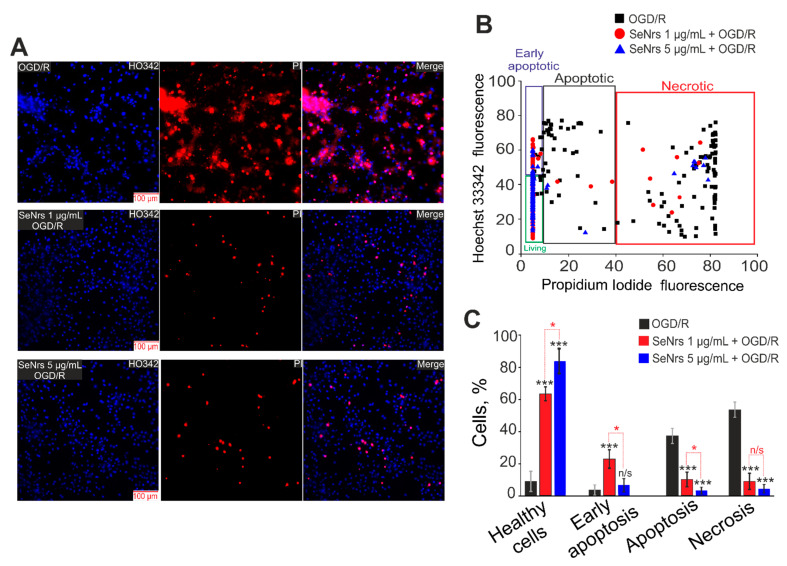
The effect of 24-h pre-incubation of cortical astrocytes with 1 or 5 μg/mL of SeNrs on the OGD/R-induced cell death. (**A**) Double staining of cells with Hoechst 33342 (HO342), propidium iodide (PI), merge HO342 with PI. OGD/R—induction of OGD (2 h) and reoxygenation (24-h) without preincubation with SeNrs. (**B**) Cytogram demonstrating the viability of cortical astrocytes after OGD/R (2h and 24-h reoxygenation) and after 24-h pre-incubation with 1 or 5 μg/mL SeNrs and OGD/R. *X*-axis—the intensity of PI fluorescence; *Y*-axis—the intensity of Hoechst 33342 fluorescence. Cells were stained with the probes 24-h after the OGD/R. (**C**) Effect of 24-h pre-incubation with 1 or 5 μg/mL SeNrs on the induction of necrosis and apoptosis. The number of replicates for cell cultures was 3, the number of cover-slips with cells for each sample was 5. Statistical significance was assessed using *t*-tests. Comparison of experimental groups vs OGD/R: n/s—data not significant (*p* > 0.05), * *p* < 0.05, *** *p* < 0.001. Comparison of experimental groups with each other is indicated in red.

Preincubation of cortical astrocytes with SeNrs at concentrations of 1 µg/mL or 5 µg/mL led to the suppression of apoptosis and necrosis in a more toxic model of ischemia-OGD for 2 h followed by 24-h reoxygenation-(OGD/R) (Figure 3). OGD/R caused the induction of late stages of apoptosis (cells are characterized by a high level of Hoechst 33342 fluorescence simultaneously with the appearance of some membrane permeability to propidium iodide (PI)) in 40% of astrocytes (Figure 3A,B) and the appearance of necrosis (high level of PI fluorescence) in 60% or more astrocytes (Figure 3C).

Additional analysis of the effects of SeNrs on the induction of necrosis and apoptosis was performed using the Apoptosis/Necrosis Detection Kit (ab176750, Abcam, Cambridge, UK) (Supplementary Figure S2). It turned out that 24-h after OGD/R, necrosis was registered in 62% of cells, and apoptosis in 27% of astrocytes. Preincubation of astrocytes with SeNrs at 1 µg/mL or 5 µg/mL dose-dependently reduced apoptosis to 20% and 14% of cells, and necrosis to 14% and 10% of cells, respectively (Supplementary Figure S2B).

**Figure 4:**
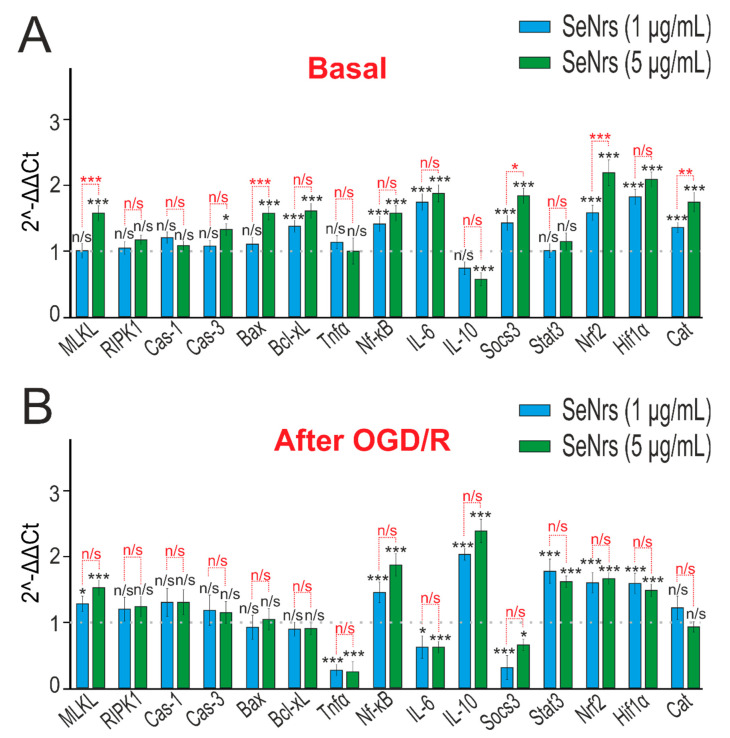
The effect of 24-h incubation of mice cortical astrocytes with 1 μg/mL or 5 μg/mL SeNrs on the basal (**A**) and OGD/R-induced (**B**) expression of genes regulating cell death and inflammation pathways. Gene expression in intact cells are marked with a dashed line for panel (**A**). Gene expression in OGD/R cells without preincubation with SeNrs compounds is marked with a dashed line for panel (**B**). The results are expressed as 2^−ΔΔCt^, where (ΔΔCt) is the difference between the ΔCt values between the gene under experiment and a housekeeping gene. Statistical significance was assessed using *t*-tests. Comparison of experimental groups with control: n/s—data not significant (*p* > 0.05), * *p* < 0.05, ** *p* < 0.01, and *** *p* < 0.001, n = 3 Comparison of experimental groups with each other is indicated in red.

An analysis of the expression of genes encoding proteins that regulate cell death showed that the preincubation of astrocytes with 1 μg/mL SeNrs led to increased expression of 7 of the 15 genes studied (Figure 4A, blue columns), while 5 μg/mL SeNrs affected the expression of 11 genes (Figure 4A, green columns). SeNrs contributed to an increase in mRNA expression by more than two times for the genes encoding the *Hif1 alpha* subunit of the hypoxia-induced transcription factor and the *Nrf-2* transcription factor, which is involved in a complex regulatory network and plays a pleiotropic role in the regulation of metabolism, inflammation, autophagy, proteostasis, mitochondrial physiology, and immune responses (Figure 4A).

Preincubation of astrocytes with SeNrs and subsequent OGD/R led to an increase in the expression of IL-10, which has an anti-inflammatory effect, and simultaneously to a decrease in the level of expression of the tumor necrosis factor *Tnfα*. In addition, after preincubation of cells with SeNrs, an increased level of expression of the *Stat3*, *Nrf2*, and *Hif1 alpha* genes persisted. (Figure 4B).

**Figure 5:**
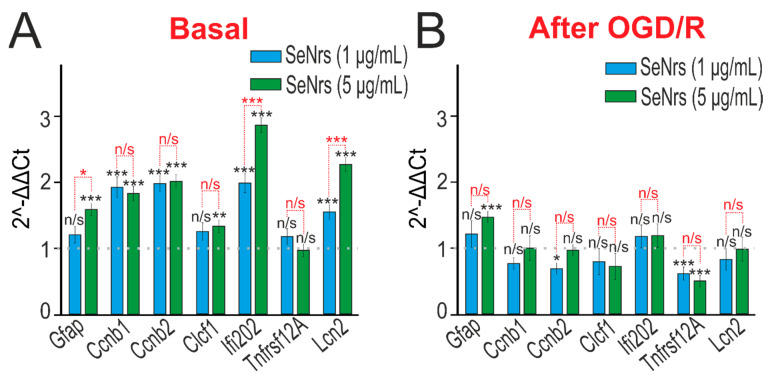
The effect of 24-h incubation of mice cortical astrocytes with 1 μg/mL or 5 μg/mL SeNrs on the basal (**A**) and OGD/R-induced (**B**) expression of genes encoding markers of reactive astrogliosis. Gene expression in intact cells is marked with a dashed line for panel (**A**). Gene expression in OGD/R cells without preincubation with SeNrs compounds is marked with a dashed line for panel (**B**). The results are expressed as 2^−ΔΔCt^, where (ΔΔCt) is the difference between the ΔCt values between the gene under experiment and a housekeeping gene. Statistical significance was assessed using *t*-tests. Comparison of experimental groups with control: n/s—data not significant (*p* > 0.05), * *p* < 0.05, ** *p* < 0.01, and *** *p* < 0.001. Comparison of experimental groups with each other is indicated in red. The number of samples is 3.

We have previously shown that spherical selenium nanoparticles activated reactive astrogliosis in neuroglial culture of the cerebral cortex [27]. PCR analysis showed that preincubation of cortical astrocytes with SeNrs led to an increase in the baseline expression of widely used markers of reactive astrocytes *GFAP* (glial fibrillary acid protein) and *Lcn2* (Lipocalin-2), proliferation genes, including late-phase cyclins b1 and b2 (*Ccnb1* and *Ccnb2*), and *Ifi202b* (interferon-activated gene 202B), which has anti-inflammatory activity (Figure 5A).

Preincubation of astrocytes with SeNrs and subsequent OGD/R led to an increase in *GFAP* expression and suppression of expression of *TNFRSF12A* (TNF receptor superfamily member 12A), which is involved in inflammatory processes in astrocytes (Figure 5B).

**Figure 6:**
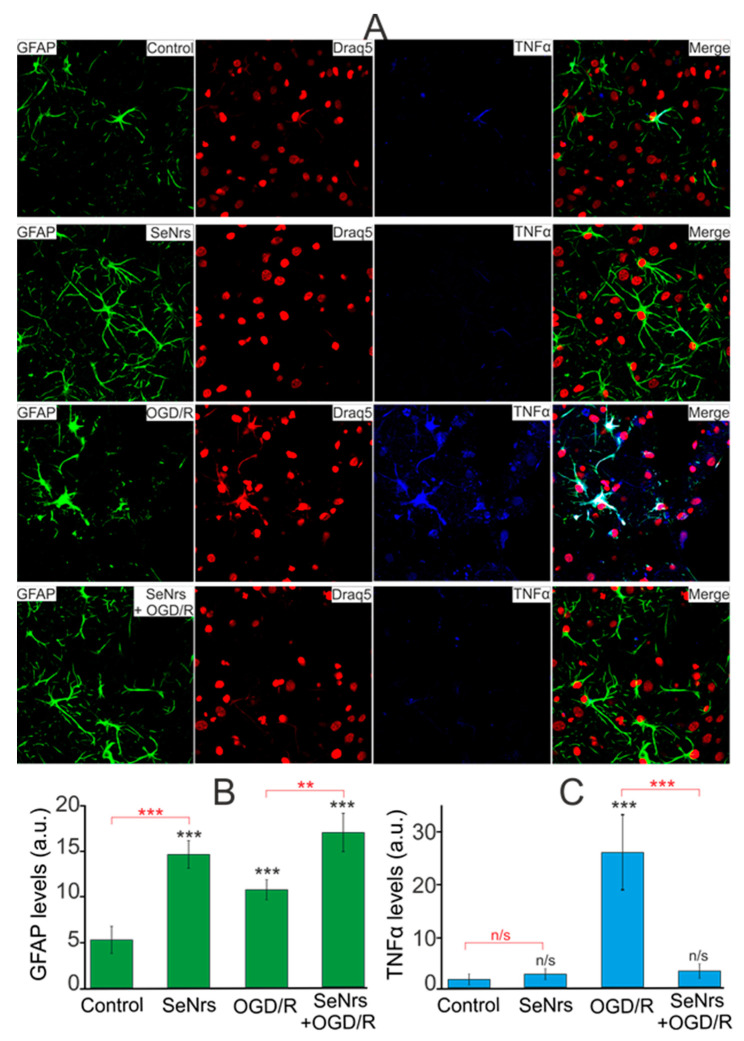
Effect of 24-h preincubation of cortical astrocytes with 5 μg/mL SeNrs on baseline and OGD/R induced levels of tumor necrosis factor-alpha (TNFα) expression. (**A**) Immunostaining of glial fibrillary acidic protein (GFAP, green) and TNFα (blue) in cortical astrocytes in the control, after 24-h of incubation with 5 μg/mL SeNrs, after OGD/R and OGD/R after 24-h of incubation with SeNrs. Draq5 nuclei staining (red). (**B**,**C**) Intensity levels of GFAP (**B**) and TNFα (**C**) were determined by confocal imaging. We analyzed individual cells that had fluorescence of secondary antibodies. The quantitative data reflecting the level of GFAP or TNFα expression are presented as fluorescence intensity values in summary bar charts (mean +/− SEM). The values were averaged by 200 cells for each column. The results obtained after immunostaining agree well with the data of fluorescence presented in panel (**A**). Each value is the mean ± SE (n ≥ 3, *p* < 0.05). Statistical significance was assessed using paired *t*-tests. Comparison with control, *** *p*-level < 0.001, ** *p* < 0.01, n/s—data not significant (*p* > 0.05).

The results of PCR analysis were confirmed by immunocytochemical staining of astrocytes simultaneously with anti-GFAP and TNFα antibodies (Figure 6A). The results of image analysis using the ImageJ software (https://imagej.net/ij/, accessed on 6 July 2023) package showed that in the control there was practically no fluorescence of secondary antibodies against TNFα (Figure 6C—Control), with a moderate fluorescence of secondary antibodies against GFAP (Figure 6B—Control), reflecting the level of the studied intracellular proteins. Preincubation of astrocytes with 5 μg/mL SeNrs resulted in an increase in the fluorescence level of anti-GFAP antibodies (Figure 6A,B) without a significant increase in the level of fluorescence of anti-Tnfα antibodies (Figure 6C, SeNrs). Staining of astrocytes 24-h after OGD/R showed increased levels of GFAP (Figure 6B—OGD/R) and TNFα (Figure 6C—OGD/R) compared with the control. However, OGD/R after preincubation of astrocytes with 5 μg/mL SeNrs resulted in an increase in the level of GFAP protein (Figure 6B—SeNrs + OGD/R) with a decrease in the level of Tnfα (Figure 6C—SeNrs + OGD/R) compared with the experimental group astrocytes subjected to OGD/R alone. These immunocytochemical staining data correlate well with the results of PCR analysis of OGD/R-induced expression of genes involved in cell death (Figure 4) and the activation of reactive astrogliosis (Figure 5).

**Figure 7:**
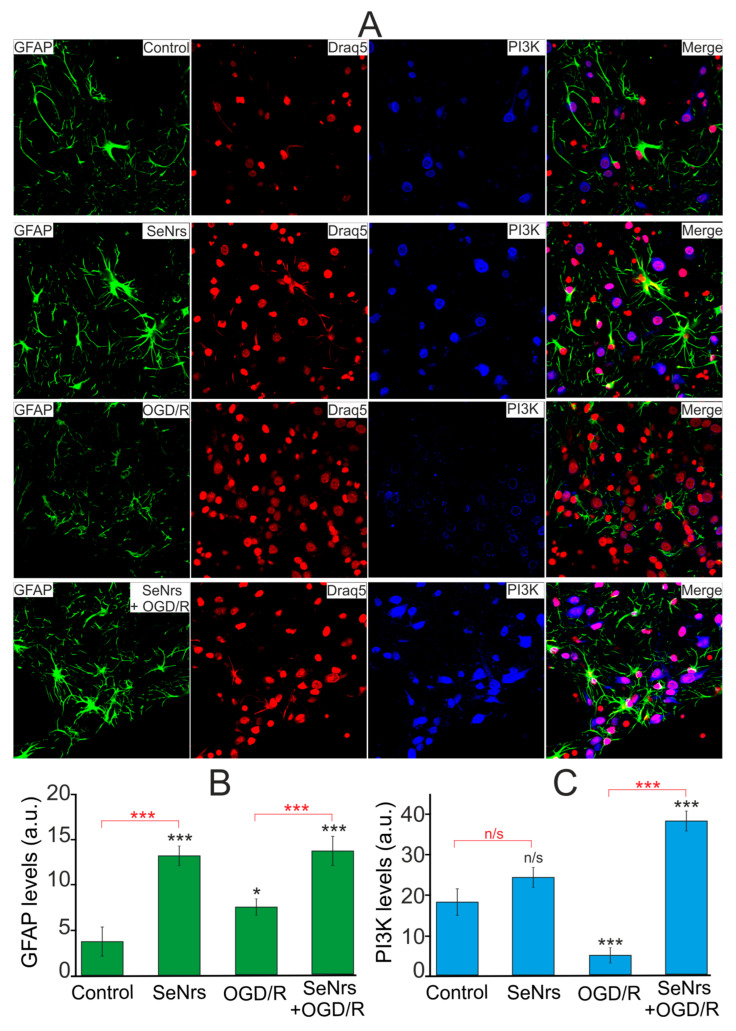
Effect of 24-h pre-incubations of cortical astrocytes with 5 μg/mL SeNrs on baseline and OGD/R induced levels of phosphoinositide 3-kinase (PI3K) expression. (**A**) Immunostaining of glial fibrillary acidic protein (GFAP, green) and PI3K (blue) in cortical astrocytes in the control, after 24-h of incubation with 5 μg/mL SeNrs, after OGD/R and OGD/R after 24-h of incubation with SeNrs. Draq5 nuclei staining (red). (**B**,**C**) Intensity levels of GFAP (**B**) and PI3K (**C**) were determined by confocal imaging. We analyzed individual cells that had fluorescence of secondary antibodies. The quantitative data reflecting the level of GFAP or PI3K expression are presented as fluorescence intensity values in summary bar charts (mean +/− SEM). The values were averaged by 200 cells for each column. The results obtained after immunostaining agree well with the data of fluorescence presented in panel A. Each value is the mean ± SE (n ≥ 3, *p* < 0.05). Statistical significance was assessed using paired *t*-tests. Comparison with control, *** *p*-level < 0.001, * *p*-level < 0.05, n/s—data not significant (*p* > 0.05).

Phosphoinositide 3-kinase is known to be involved in the activation of the defense mechanisms of brain cells [29,30,31]. Immunocytochemical staining of cortical astrocytes with antibodies against GFAP and PI3K showed (Figure 7) that the preincubation of astrocytes for 24-h with 5 μg/mLSeNrs caused an increase in the level of not only GFAP (Figure 7B—SeNrs), but also the level of PI3K (Figure 7—SeNrs). Whereas after OGD/R, on the contrary, there was a decrease in the number of cells stained with antibodies against PI3K, as well as the level of antibody fluorescence in cells (Figure 7C, OGD/R). In contrast, the preincubation of astrocytes with SeNrs contributed not only to an increase in the GFAP level (Figure 7B, SeNrs + OGD/R), but also to an increase in the content of PI3K (Figure 7C, SeNrs + OGD/R) after OGD/R.

Thus, preincubation of cortical astrocytes with SeNrs suppresses the processes of necrosis and apoptosis caused by ischemia/reoxygenation conditions. Such a protective effect of selenium nanorods occurs due to an increase in the expression level of key genes encoding anti-apoptotic, anti-inflammatory proteins, and transcription factors. This activates the process of reactive astrogliosis, which correlates with an increase in the level of the protein phosphoinositol-3-kinase and a decrease in the level of TNFα.

### 2.4. The Induction of Reactive Astrogliosis and the Cytoprotective Effects of SeNrs Are Abolished upon Inhibition of Endocytosis

**Figure 8:**
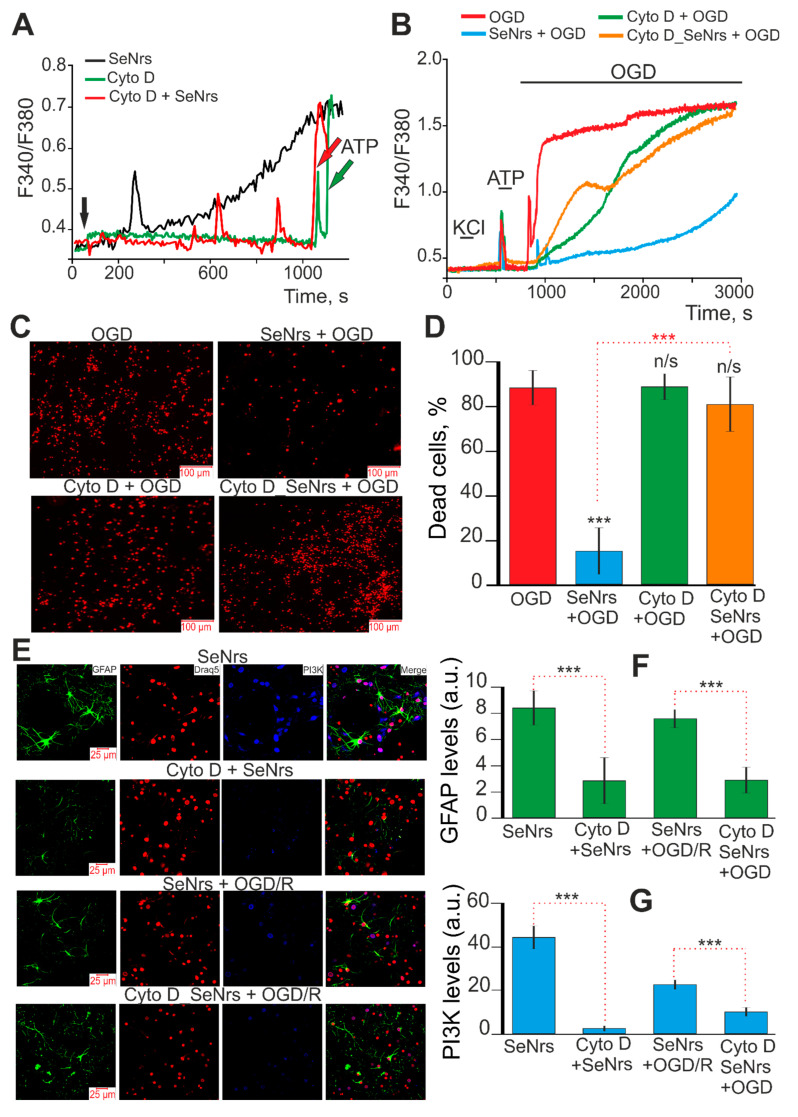
The effect of an inhibitor of actin-dependent pathways of endocytosis (Cytochalasin D, Cyto D, 10 µM) on astrocyte Ca^2+^ signals upon application of SeNrs (5 μg/mL) (**A**), on the cytoprotective effects of SeNrs (5 μg/mL) upon application oxygen-glucose deprivation (OGD) (**B**–**D**), activation of reactive astrogliosis (**E**,**F**), and expression of phosphoinositide 3-kinase (PI3K) (**E**,**G**). (**A**) Ca^2+^ signals of cortical astrocytes upon application of 5 μg/mL SeNrs (black curve), 10 μM Cytochalasin D (Cyt D, green curve), and 5 μg/mL SeNrs after 2-h preincubation of cells with 10 μM Cytochalasin D (Cyto D + SeNrs, red curve). Averaged Ca^2+^ signals for several tens of astrocytes are shown. At the end of the experiment, 10 μM ATP was added to the cells. (**C**) Effects of 24-h preincubation of cortical astrocytes with Cytochalasin D (10 µM) on OGD-induced Ca^2+^ signals. At the beginning of the experiment, a short-term application of 35 mM KCl and 10 µM ATP was added to detect neurons and astrocytes, respectively. F340/F380—The ratio of Fura-2 fluorescence in the 340 nm registration channel to the 380 nm fluorescence registration channel. (**C**,**D**) Effects of 24-h preincubation of cortical astrocytes with Cytochalasin D (10 µM) on OGD-induced cell death. Astrocyte culture staining after 40 min OGD with propidium iodide (**C**) and necrotic death analysis (**D**). Abbreviations for panels (**B**–**D**) OGD—oxygen-glucose deprivation for 40 min; SeNrs + OGD—astrocytes were preincubated for 24-h with 5 μg/mL SeNrs and then OGD was created; Cyto D + OGD—cells were preincubated for 24-h with 10 µM Cytochalasin D and then OGD was created; Cyto D_SeNrs + OGD—astrocytes were preincubated for 2 h with 10 µM Cytochalasin D and then 5 µg/mL SeNrs were added for 24-h (Cyto D was maintained in the culture medium for 24-h) and then OGD was created. (**E**–**G**) Effects of 24-h preincubation of cortical astrocytes with Cytochalasin D (10 µM) on SeNrs and OGD/R-induced astrocyte reactivation (**E**,**F**) and PI3K expression (**E**,**G**). (**E)** Immunostaining of glial fibrillary acidic protein (GFAP, green) and PI3K (blue) in cortical astrocytes after 24-h of incubation with 5 μg/mL SeNrs (SeNrs group), after 24-h of preincubation with 10 μM Cytochalasin D and 5 μg /mL SeNrs (Cyto D + SeNrs group), after 24-h of incubation with 5 µg/mL SeNrs and OGD/R (SeNrs + OGD/R group), and after 24-h of preincubation with 10 µM Cytochalasin D with 5 µg/ mL SeNrs and OGD/R (Cyto D_SeNrs + OGD/R group). Draq5 nuclei staining (red). (**F**,**G**) Intensity levels of GFAP (**F**) and PI3K (**G**) were determined by confocal imaging. We analyzed individual cells that had fluorescence of secondary antibodies. The quantitative data reflecting the level of GFAP or PI3K expression are presented as fluorescence intensity values in summary bar charts (mean +/− SEM). The values were averaged by 150–200 cells for each column. Each value is the mean ± SE (n ≥ 3, *p* < 0.05). Statistical significance was assessed using paired *t*-tests. Comparison with control, *** *p*-level < 0.001, n/s—data not significant (*p* > 0.05).

It is known that Cytochalasin D blocks or largely suppresses all actin-dependent pathways of endocytosis, depending on the duration of incubation (from 2 to 24-h) [32,33]. Preincubation of astrocytes with 10 μM Cytochalasin D (Cyto D) for 2-h leads to complete suppression of Ca^2+^ responses to SeNrs application (Figure 8A, red curve). Astrocytes did not respond with “standard“ Ca^2+^ signals to SeNrs application in the form of a gradual increase in [Ca^2+^]_i_ (Figure 8A, black curve), but single Ca^2+^ pulses were recorded. Application of 10 μM Cyto D in an acute experiment did not cause the generation of Ca^2+^ signals by astrocytes (Figure 8A, green curve). At the same time, the addition of ATP (10 μM) at the end of the experiments indicates the functionality of the Ca^2+^ signaling system of astrocytes.

After a 24-h preincubation of astrocytes with 5 μg/mL SeNrs, the OGD-induced increase in [Ca^2+^]_i_ was suppressed (Figure 8B, blue curve) and cell death was reduced to 17% (Figure 8C,D, SeNrs + OGD). After a 2-h preincubation of astrocytes with Cyto D SeNrs was added to culture medium at a concentration of 5 μg/mL for 24-h. After this preincubation, OGD induced a biphasic increase in [Ca^2+^]_i_ (Figure 8B, orange curve) which was comparable with OGD experimental group (Figure 8B, red curve). Cell death after OGD was 83% of cells (Figure 8C,D—OGD group), and after preincubation with SeNrs together with Cyto D, necrosis was recorded in 81% of astrocytes (Figure 8C,D—Cyto D_SeNrs + OGD). Preincubation of astrocytes with 10 μM Cyto D for 24-h did not suppress the OGD-induced increase in [Ca^2+^]_i_ (Figure 8B—green curve) and did not reduce astrocyte death (82%) (Figure 8C,D—Cyto D + OGD).

Immunocytochemical staining of astrocytes with antibodies against GFAP and PI3K showed that the effect of 24-h preincubation with SeNrs on the increase in the expression of these proteins was abolished after preincubation with Cyto D (Figure 8E–G—Cyto D + SeNrs). Similarly, after preincubation of asrocytes with SeNrs and subsequent OGD/R, activation of reactive astrogliosis occurred, recorded by an increase in the level of GFAP and PI3K (Figure 8E–G—SeNrs + OGD/R). However, preincubation of astrocytes with Cyto D abolished this SeNrs-induced increase in GFAP and PI3K expression (Figure 8E–G—Cyto D_SeNrs + OGD/R).

Thus, the SeNrs-induced activation of reactive astrogliosis and the cytoprotective effects of SeNrs during OGD and reoxygenation occur due to endocytosis of nanoparticles into astrocytes and activation of the Ca^2+^ signaling system of cells. In the absence of these two components, the protective effects of SeNrs were completely abolished.

## 3. Discussion

In addition to the development and study of the mechanisms of action of spherical nanoparticles of various origins, in recent years, active work has been carried out on the synthesis of nanostructures of various shapes and the study of their physiological effects. Research is underway on various types of scaffolds, including electrospun nanofibers, which can be an effective substrate for neuron differentiation, axon growth, and the formation of new cell–cell and cell–matrix interactions [34,35]. Nanofibers may be a platform for the selective and controlled release of drugs to brain cells, protecting them from damage [36]. Carbon nanotubes have been developed that have shown their electrically conductive capacity, strong mechanical properties, and morphological similarity to neurites [37]. The great advantage of nanotubes is that they are highly flexible, very strong, and can be easily modified with active agents [38,39]. It has been shown that carbon nanotubes can be widely used as bone implants and for the treatment of rheumatoid arthritis or osteoporosis [38,40], while the use of these nanostructures in neuroprotection has not been practically studied. Using carbon nanotubes as an example, it has been shown that they penetrate cells through 2 pathways, active endocytosis, and passive nano penetration [41]. The use of nanotubes for the treatment of neurodegenerative diseases depends on their physicochemical properties, origin, and method of preparation. At the same time, their large surface area, ability to be easily modified with drug molecules, and biocompatibility with the neural system suggests a high efficiency of nanotubes [42].

Nanostructures in the form of filaments can enhance the ability of neurons to attach to the substrate and the ability of their neurites to grow [43]. Scaffolds grown from nanofilaments are able to improve their integration into the Parkinsonian brain and improve the re-innervation of the striatum of Parkinsonian mice, especially when these nanostructures have been doped with GDNF [44]. Loading nanofilaments with drugs showed that nanofilaments started to float immediately with zero floating lag time; the formulations did not sink after 24-h. The floating nanofilaments showed a lower first release between 20% and 57% when compared to non-floating nanofilaments (40–82% within 2 h). This indicates that drugs that should be administered frequently might be taken orally ’once a day’ and improve patient compliance [45].

One-dimensional (1D) nanostructural materials such as nanotubes, nanowires, nanorods, and nanothreads have recently received much attention due to their interesting properties and are potential candidates for application in different fields. The one-dimensional nanostructured selenium nanowires have been widely studied because of their functional properties in superconductivity [46], high photoconductivity, and catalytic activity. Selenium nanowires have field application in the areas of photoelectric cells, xerography, light-measuring devices, and solar batteries [47]. Similar physical properties should be assumed for selenium nanorods.

The shape of rods or tubes have pronounced properties to remain in the body for a long time and exert their targeted effects on tissues. It is shown that the residence time of long rod nanoparticles and short rod silica nanoparticles in the gastrointestinal tract is significantly higher compared to spherical nanoparticles [9,48,49]. It has also been found that oral administration of rod-shaped silica nanoparticles leads to a greater accumulation of Si in the liver and kidney. It was also found that the short rod nanoparticles reached a higher content in all the organs at 2-h and 24-h, while the long rod nanoparticles attained a higher content in all the organs at 7 days. These results indicated that it was more difficult to remove the long rod nanoparticles from the reticule–endothelial system (RES) organs and they were protected from macrophages, resulting in a longer blood-circulation [9]. Filament-shaped particles had a longer circulation half-life in vivo compared with prototypical spherical particles after intravenous injection [50,51]. With regard to the removal of nanoparticles of various shapes from the body, clear correlations have been established. The released amounts of degradation products of long and short rods were noticeably less than the number of spherical nanoparticles 2-h after oral administration [9].

The filamentous form of nanostructures has a number of advantages for use in neuroscience. It has been shown for carbon nanotubes that the structural features and dimensions are very similar to many elements of the neuronal network (ion channels, signaling proteins, and elements of the neuronal cytoskeleton), which allows them to be used to regulate the functional activity of neuroglial networks [52]. Carbon nanotubes have been shown to enhance neuronal electrical signaling [53], decrease astrocyte formation, macrophage density [54], and have been shown to increase the differentiation of progenitor cells to neurons [55]. Interestingly, carbon threads [56] were also compatible with neural cells as demonstrated by culturing hippocampal neurons and PC12 cells on them. After a week in culture, neurite outgrowth on the nanorods surface showed neuron-specific labeling (β111 tubulin staining), indicating the biocompatibility of these nanorods for the construction of electrodes or nanowires in implantable devices [57]. All these effects on the excitation of neuronal networks undoubtedly occur due to the activation of the Ca^2+^ signaling system of neurons and astrocytes.

In our experiments, it was found that the use of SeNrs in an acute experiment led to the generation of Ca^2+^ signals of astrocytes in the form of a gradual increase in the base concentration of [Ca^2+^]_i_, which is similar in shape and amplitude to the action of spherical selenium nanoparticles (SeNPs) with a diameter of 50 nm. At the same time, the EC50 for activation of the Ca^2+^ signaling system of astrocytes under the action of SeNrs is higher (4.9 μg/mL), compared to spherical SeNPs with a diameter of 50 nm (1 μg/mL) and 400 nm (2.4 μg/mL) [28]. Thus, the shape of nanoparticles in the form of SeNrs affects the dose dependence of the activation of Ca^2+^ signals of astrocytes, which also determines the physiological effects of nanoselenium. It turned out that preincubation of cortical astrocytes with SeNrs protects them from death under the action of OGD both at a concentration of 1 µg/mL and 5 µg/mL, suppressing the global increase in [Ca^2+^]_i_ and cell necrosis.

Molecular selenium [58,59] and spherical nanoselenium [23,60,61] clearly show anti-apoptotic and anti-inflammatory effects on brain tissue. However, there are no such works for nanoselenium in the form of nanorods. At the same time, there are single works showing the protective effect of materials in the form of nanofibers on tissue.

Poly(ε-caprolactone)/polysialic acid hybrid nanofibers scaffold encapsulating glucocorticoid methyl-prednisolone has been shown to suppress TNF-α and interleukin-6 IL-6 release by inhibiting ionized calcium-binding adapter molecule 1 (Iba1)-positive microglia/macrophage activation and reduces apoptosis-associated Caspase-3 protein expression in the spinal cord injury model [62]. In addition, the wound healing effects of structures in the form of nanofibers have been established. Ag-nanofibers upregulated molecules implicated in the TGF-β1/Smad signaling pathway, hence boosting collagen synthesis and improving wound healing by upregulating TGF-β1 secretion and activating the TGF-β1/Smad signaling pathway during the early stages of wound healing, boosting the adhesion and proliferation of fibroblasts [63].

Nanoselenium in the form of nanorods caused an increase in the baseline and OGD/R-induced expression of the antioxidant gene *Nrf2*, anti-inflammatory interleukin 10 (*IL-10*), and *Hif2α*. In this case, the expression and content of TNFα in the cortical astrocytes was suppressed, which was expressed in the inhibition of necrosis and apoptosis caused by ischemia/reoxygenation. Along with the suppression of apoptosis and inflammation, SeNrs led to the activation of reactive astrogliosis, which was detected by increased expression of genes encoding protein markers of this process. It is known that reactive astrogliosis is a reaction of astrocytes to pathological conditions including trauma, brain aging, ischemia, bacterial or virus infections, and various neurodegenerative diseases [64,65].

For a long time, it was believed that reactive astrogliosis has an extremely detrimental effect on the nervous system in the form of scar formation, which prevented the restoration of neurotransmission and brain functions after damage [65]. It is known that the reactivation of astrocytes is a complex stepwise process leading to both negative and positive effects on the brain, depending on the severity of the injury. In reactive astrogliosis, various changes occur in the patterns of gene expression of proteins responsible for the induction of cell proliferation and hypertrophy, which only in severe cases lead to the formation of scars. Moreover, there is strong evidence for the beneficial effects of scar formation as it can limit brain tissue damage and inflammation [64,66,67]. It is customary to subdivide reactive astrocytes into A1 and A2 types. Reactive A1 astrocytes are known to have lost many of the characteristic functions of astrocytes, including promoting neuron survival and growth, synapse formation and function, and the ability to phagocytize synapses and myelin debris. While A2, which is induced by ischemia [68], strongly promotes neuronal survival and tissue repair [69,70]. As our experiments showed, 24-h exposure to SeNrs led to an increase in the baseline expression of genes that encode proteins of the A2 type of reactive astrogliosis. After ischemia/reoxygenation, the effects of SeNrs were observed only for *GFAP*, the level of which increased in astrocytes, however, this increase coincided with the suppression of the expression of *Tnfrsf12A* involved in inflammation. The role of reactive astrocytes in neuroprotection is difficult to overestimate. A2-reactive astrocytes have been shown to be able to secrete a wide set of neuroprotective molecules. For example, they exert their anti-oxidative functions by secreting molecules such as glutathione [71]. They also secrete a wide array of neurotrophic factors such as glia-derived neurotrophic factor (GDNF), ciliary neurotrophic factor (CTNF), brain-derived neurotrophic factor (BDNF), and nerve growth factor (NGF), aiding the growth and survival of neurons [72].

Interestingly, reactive astrogliosis occurred simultaneously with an increase in the intracellular content of phosphoinositide 3-kinase (PI3K) in astrocytes both after preincubation with SeNrs and after preincubation with SeNrs followed by ischemia/reoxygenation. It is known that reactive astrocytes displayed pro-inflammatory adaptability through Notch-PI3K-AKT signaling activation in response to inflammatory stimulation [73]. We have previously shown that spherical nanoparticles exert their effect on human glioblastoma cells through the activation of the Ca^2+^ signaling system and the opening of Connexin43 (Cx43) hemichannels, which led to the induction of controlled death of cancer cells [74]. At the same time, the same signaling cascade was realized in normal astrocytes, but it had a neuroprotective effect during ischemia [27]. It can be assumed that a similar signaling pathway is also implemented in the case of the use of SeNrs. It is known that mRNA and protein levels of PI3K, AKT, as well as Cx43, were elevated in reactive astrocytes from normal rats [73,75,76,77]. Thus, reactive astrogliosis induced by preincubation with SeNrs is closely related to the Ca^2+^ signaling system of astrocytes, an increase in the PI3K level, and suppression of cell death under the action of ischemia and reoxygenation.

A possible explanation for the high survival of astrocytes due to OGD/R after preincubation with SeNrs could be NF-κB activation. This transcription factor is located at the intersection of many intracellular signaling cascades, among which the following should be noted: cascades involving cAMP, TNFα, IL-1, and the PI3K-Akt signaling pathway [78,79,80]. A decrease in TNFα expression (including a decrease in the amount of protein) shown in our experiments is consistent with the data obtained on immune cells [81]. This decrease correlates with the induction of reactive astrogliosis. It has been established that the activation of astrocytes plays a protective role during neurodegenerative processes resulting from inflammatory processes in the hippocampus during ischemia, reducing the level of TNFα [82] and increasing the level of PI3K, as we have shown. The TNFα and PI3K-Akt signaling pathways have in common that NF-kB is activated [83]. Therefore, most likely, the contribution of TNFα to the activation of NF-kB under the action of SeNrs is minimal, while the PI3K-Akt cascade may be more involved in this process. As shown by our experiments, an increase in [Ca^2+^]_i_ in astrocytes upon application of SeNrs led to an increase in the expression of markers of reactive astrogliosis (primarily GFAP). Inhibition of actin-dependent endocytosis suppressed Ca^2+^ responses to SeNrs, reactive astrogliosis, and correlated with increased necrotic death of astrocytes during OGD. It is known that, firstly, an increase in [Ca^2+^]_i_ can induce reactive astrogliosis in pathological processes [84], and secondly, reactive astrogliosis is characterized by abnormal Ca^2+^ signals of astrocytes [85,86].

## 4. Materials and Methods

Experimental protocols were approved by the Bioethics Committee of the Institute of Cell Biophysics. Experiments were carried out according to Act708n (23 August 2010) of the Russian Federation National Ministry of Public Health, which states the rules of laboratory practice for the care and use of laboratory animals, and the Council Directive 2010/63 EU of the European Parliament on the protection of animals used for scientific purposes.

### 4.1. Preparation and Characterization of Selenium Nanorods

Selenium nanorods were obtained by laser ablation of solid target and subsequent laser fragmentation of obtained Se nanoparticles in propanol-2. Resulting Se nanorods were transferred to deionized water with a resistivity of 20 MΩcm. At the first stage, a solid target of Se was placed at the bottom of the cuvette under a thin layer of chemically pure propanol-2 (layer thickness was no more than 2–3 mm). The target was irradiated with a laser beam through a thin layer of propanol-2 (λ = 1064 nm; T = 4 ns; f = 1 kHz; P = 20 W; Ep = 2 mJ). The beam was moved on the target surface along a given trajectory in the form of parallel straight lines inscribed in a rectangle using an LScanH galvanomechanical scanner (Ateko-TM, Moscow, Russia) and F-Theta lens. The typical time of ablation was 30 min.

At the second stage, the colloid of Se nanoparticles in propanol-2, which was obtained as a result of ablation, was additionally irradiated with same laser parameters in the absence of solid target during 1 h.

At the final stage, Se nanoparticles were transferred in deionized water by centrifugation and washing in various chemical solvents. The washing process is necessary to purify the solution from the decomposition products of propanol-2 molecules forming as a result of optical breakdown.

Initially, SeNrs nanoparticles were precipitated using an LMC-4200 centrifuge (Biosan, Riga, Latvia). Centrifugation took place at 4200 rpm for 15 min. Propanol-2 was taken from the solution with precipitated nanoparticles, and a chemically pure solution of carbon tetrachloride (CCl4) was poured instead of propanol-2. The resulting solution was placed in an ultrasonic bath (ultrasonic power was 20 W) for 10 min. Similar procedures for centrifugation, replacement of the solution, and the use of ultrasound were carried out for dimethyl sulfoxide (C_2_H_6_OS), chloroform (CHCl₃), and acetone (C_3_H_6_O). At the last stage, solvent was replaced by MQ-water.

**Figure 9:**
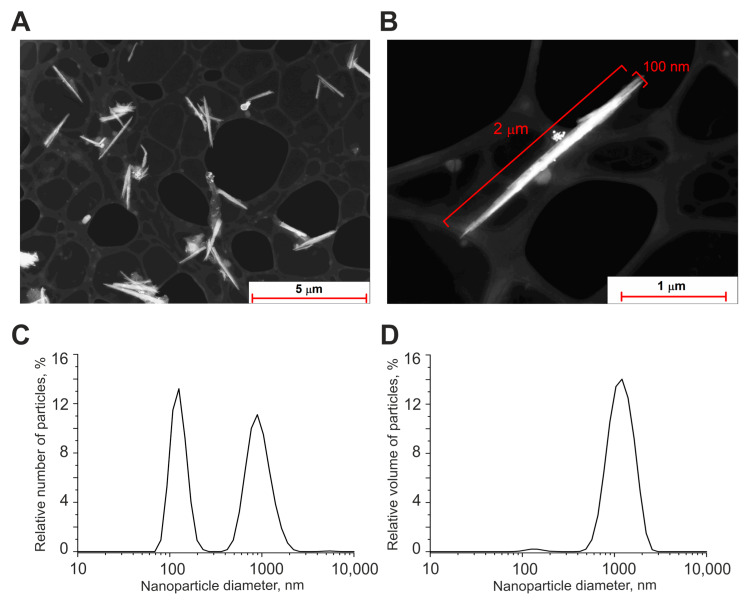
Shape and size distribution of Se nanorods. (**A**) TEM image of obtained Se nanoparticles (dark field image). (**B**) TEM image of an individual nanoparticle with a diameter of 70 nm and length of 1 µm (dark field image). (**C**) Size distribution of selenium nanoparticles. (**D**) Volume distribution of selenium nanoparticles. Data obtained using an analytical disk centrifuge and confirmed by DLS. PDI = 0.28 ± 0.5; (n = 3).

The morphology of nanoparticles was studied by a 200FE transmission electron microscope (Carl Zeiss Microscopy GmbH, Jena, Germany). According to transmission electron microscopy data, obtained Se nanoparticles have a form of nanorods (Figure 9A,B). The size of nanoparticles was characterized using a DC24000 analytical centrifuge (CPS Instruments, Prairieville, LA, USA). Nanoparticle concentration and hydrodynamic radius were evaluated using Zetasizer Ultra Red Label (Malvern, UK). It was found that the selenium nanoparticles have a bimodal size distribution (Figure 9C). The first peak of particle distribution was located at 120 nm, the half-width was in the range of 90–160 nm, the second peak of the distribution was located at 900 nm, the half-width was in the range of 600–1300 nm. The volume–size distribution of the obtained nanoparticles showed that the bulk of the volume of the nanoparticles was concentrated around the size of 1000 nm (Figure 9D).

### 4.2. Primary Astrocytes Culture

Astroglial cell cultures were isolated from the brains of 1–2-day-old rats according to the modified McCarthy and de Vellis protocol [87]. The brains were extracted, the cerebral cortex was separated, the meninges were removed, and tissue was ground and incubated in 0.05% trypsin-EDTA solution at 37 °C for 30 min. After enzymatic digestion, the tissues were washed twice in PBS and then dissociated by glass Pasteur pipette in a culture medium consisting of DMEM (PanEco, Russia), 1 g/L D-glucose, and 10% FBS (Biosera, Kansas City, MO, USA), with the addition of 2 mM glutamine (PanEco, Moscow, Russia). The suspension of cells was transferred on ventilated culture vials (Costar, Washington, DC, USA) precoated with poly-D-lysine (10 µg/mL). The cells were cultivated at 37 °C and 5% CO_2_. After 5–6 days, the cultures were subjected to vibration on an orbital shaker at 200 rpm for 16 h to detach and remove microglia. After 10 to 20 days, in vitro astrocytes were used for experiments.

### 4.3. Immunocytochemistry

To detect GFAP, PI3K, and TNFα in astrocytes, we used an immunocytochemical assay [27,28]. The cells were fixed with 4% paraformaldehyde +0.25% glutaraldehyde in PBS for 20 min and washed three times with ice-cold PBS for 5 min. Glutaraldehyde was added into the fixative solution to minimize the washing of antibodies from cells during permeabilization. To permeabilize cells, we used 0.1% Triton X-100 solution for 15 min. Fixed cells were incubated in 10% donkey serum for 30 min at room temperature to block non-specific antibody binding sites. The cells were then incubated with primary antibodies against investigated proteins for 12 h at 4 °C. The fixed cells were subsequently washed with PBS (3 times for 5 min) and probed with secondary antibodies conjugated with a fluorescent label. We used purified anti-GFAP antibody (BioLegend, RRID: AB_2632644), purified rabbit monoclonal antibody to PI3-Kinase p85 alpha ([EPR18702], (ab191606)), polyclonal anti-TNFα goat antibodies (sc-1351, Santa Cruz Biotechnology, Dallas, Texas, USA), donkey polyclonal secondary antibody to rabbit IgG (H+L) (Alexa Fluor-647) (Jackson ImmunoResearch Europe LTD, Cambridge, UK, RRID: AB_2492288), and donkey polyclonal secondary antibody to mouse IgG-H&L (Alexa Fluor-594) (Abcam, Cambridge, UK, RRID: AB_2732073). Dilutions of primary and secondary antibodies were performed according to the manufacturer’s recommendations for immunocytochemical staining. The fluorescence of antibodies was visualized with an inverted confocal microscope Leica TCS SP5 (Leica, Wetzlar, Germany). Registration of the secondary antibodies’ fluorescence for the control and experimental groups of cell cultures was carried out at the same microscope setting.

### 4.4. Fluorescent Ca^2+^ Measurements

To detect the changes in [Ca^2+^]_i_, cell cultures were loaded with Fura-2 (4 µM; 40 min incubation; 37 °C). The cells were stained with the probe dissolved in Hank’s balanced salt solution (HBSS) composed of (mM): 156 NaCl, 3KCl, 2MgSO_4_, 1.25KH_2_PO_4_, 2CaCl_2_, 10 glucose, and 10 HEPES, pH 7.4. To measure [Ca^2+^]_i_, we used the system based on an inverted motorized microscope Leica DMI6000B with a high-speed monochrome CCD-camera HAMAMATSU C9100. For excitation and registration of Fura-2 fluorescence, we used FU-2 filter set (Leica, Wetzlar, Germany) with excitation filters BP340/30 and BP387/15, beam splitter FT-410, and emission filter BP510/84. Illuminator Leica EL6000 with a high-pressure mercury lamp as a source of excitation light. All the Ca^2+^ signals are presented as 340/380 ratio of Fura-2 fluorescence [27].

### 4.5. The Technique for Simulation of Ischemia-Like Conditions

Ischemia-like conditions (oxygen-glucose deprivation, OGD) were obtained by omitting glucose (HBSS medium without glucose) and by displacement of dissolved oxygen with argon in the leak-proof system [88]. The level of oxygen in the medium was measured using a Clark electrode. Oxygen tensions reached values of 30–40 mm Hg or less within 20 min after the beginning of displacement. Ischemia-like conditions lasting for 40 min or 2 h were created by supplying the oxygen-glucose deprivation (OGD) medium into the chamber with cultured cortical astrocytes. Constant argon fed into the experimental chamber was used to prevent the contact of the OGD medium with the atmospheric air.

### 4.6. Assessment of Cell Viability

Propidium iodide (1 µM) was used to evaluate the number of dead cells in the cell cultures before and after OGD. The cells were stained for 5 min with the probes diluted in HBSS and then rinsed with HBSS. Fluorescence of the probes was detected with an inverted fluorescent microscope Zeiss Axio Observer Z1 (Carl Zeiss, Oberkochen, Germany) using Filter Set 20. Cell death induced by OGD was assessed by propidium iodide staining (PI, 1 µM) before and after the exposures in the same microscopic field. Furthermore, we used the Ca^2+^ signals (presence or absence of a global increase in [Ca^2+^]_i_ during OGD) as an additional indicator of cell viability [88,89].

Hoechst 33342 (2 µM) and propidium iodide (1 µM) were used to evaluate the number of dead cells in the cell cultures before and after 2 h OGD and 24-h reoxygenation (OGD/R conditions). The cells were stained for 5 min with the probes diluted in HBSS and then rinsed with HBSS. Fluorescence of the probes was detected with an inverted fluorescent microscope Zeiss Axio Observer Z1 using Filter Set 01 and Filter Set 20. Discrimination of early and late apoptotic cells was performed according to the previously described method [89,90]. Five different areas of each cell culture were analyzed. Each experimental group consisted of three cell cultures from different passages.

To simultaneously monitor apoptotic and healthy cells after SeNrs treatment and OGD/R with fluorescence microscope, an Apoptosis/Necrosis Detection Kit (ab176750, Abcam) was used [28]. Cells were washed 1–2 times and resuspended with Assay Buffer. To detect apoptotic cells, Apopxin Green Indicator was used. Apoptotic cells were visualized using the FITC channel (Ex/Em = 490/525 nm). For staining necrotic cells, we used 7-aminoactinomycin D (Ex/Em = 550/650 nm). To detect healthy cells, CytoCalcein 450 was used and cells were visualized using the violet channel (Ex/Em = 405/450 nm).

### 4.7. Extraction of RNA

Mag Jet RNA Kit (Thermo Fisher Scientific, Waltham, MA, USA) was used for the extraction of total RNA [91]. The RNA quality was estimated by electrophoresis in the presence of 1 μg/mL ethidium bromide (2% agarose gel in Tris/Borate/EDTA buffer). The concentration of the extracted RNA was determined with NanoDrop 1000c spectrophotometer. RevertAid H Minus First Strand cDNA Synthesis Kit (Thermo Fisher Scientific, Waltham, MA, USA) was used for reverse transcription of total RNA.

### 4.8. Real-Time Polymerase Chain Reaction (RT-qPCR)

Each PCR was performed in a 25 μL mixture composed of 5 μL of qPCRmix-HS SYBR (Evrogen, Moscow, Russia), 1 μL (0.2 μM) of the primer solution, 17 μL water (RNase-free), 1 μL cDNA. A Dtlite Real-Time PCR System (DNA-technology, Moscow, Russia) was used for amplification. The amplification process consisted of the initial 5 min denaturation at 95 °C, 40 cycles of 30 s denaturation at 95 °C, 20 s annealing at 60–62 °C, and 20 s extension step at 72 °C. The final extension was performed for 10 min at 72 °C. All the sequences were designed with FAST PCR 5.4 and NCBI Primer-BLAST software (https://www.ncbi.nlm.nih.gov/tools/primer-blast/primertool.cgi, accessed on 6 July 2023). The data were analyzed with Dtlite software (https://dna-technology.com/sites/default/files/dtprime_dtlite_v06_part_2.pdf, accessed on 6 July 2023) (DNA-technology, Moscow, Russia). The expression of the studied genes was normalized to gene encoding Glyceraldehyde 3-phosphate dehydrogenase (GAPDH) [92,93]. Data were analyzed using Livak’s method [94].

### 4.9. Statistical Analysis

All presented data were obtained from at least three cell cultures. All values are given as the mean ± standard error (SEM) or as individual cellular signals in experiments. Statistical analyses were performed by paired *t*-test. Differences are significant * *p* < 0.05, ** *p* < 0.01, and *** *p* < 0.001. n/s—data not significant (*p* > 0.05). MS Excel, ImageJ, Origin 2016 (OriginLab, Northampton, MA, USA), and Prism GraphPad 7 (GraphPad Software, RRID: SCR_002798) software was used for data and statistical analysis.

## 5. Conclusions

Selenium nanorods showed their high cytoprotective ability in the ischemia/reoxygenation model by suppressing pro-inflammatory and pro-apoptotic signaling pathways by acting through the regulation of calcium signaling of cortical astrocytes. Selenium nanorods induce baseline reactivity of A2-type astrocytes and, as a result, an increase in the level of phosphoinositide-3-kinase and suppression of the level of TNFα. Actin-dependent transport of nanoparticles into astrocytes and activation of the Ca^2+^ signaling system play a key role in the activation of reactive astrogliosis. This new type of selenium nanoparticle represents a therapeutic tool to combat stroke, but its delayed effects in in vivo models require further research.

## Data Availability

The data presented in this study are available on request from the corresponding author.

## Supplementary Materials

The following supporting information can be downloaded at: https://www.mdpi.com/article/10.3390/ijms241512217/s1.
