# Supplementary material for: Pilot Study of Cytoprotective Mechanisms of Selenium Nanorods (SeNrs) under Ischemia-like Conditions on Cortical Astrocytes
Source: Int J Mol Sci. 2023 Jul 30;24(15):12217. doi: 10.3390/ijms241512217 (PMC10419292; doi:10.3390/ijms241512217)
Supplement: Supplementary file 1 [file ijms-24-12217-s001.zip › ijms-2520838-supplementary.pdf]

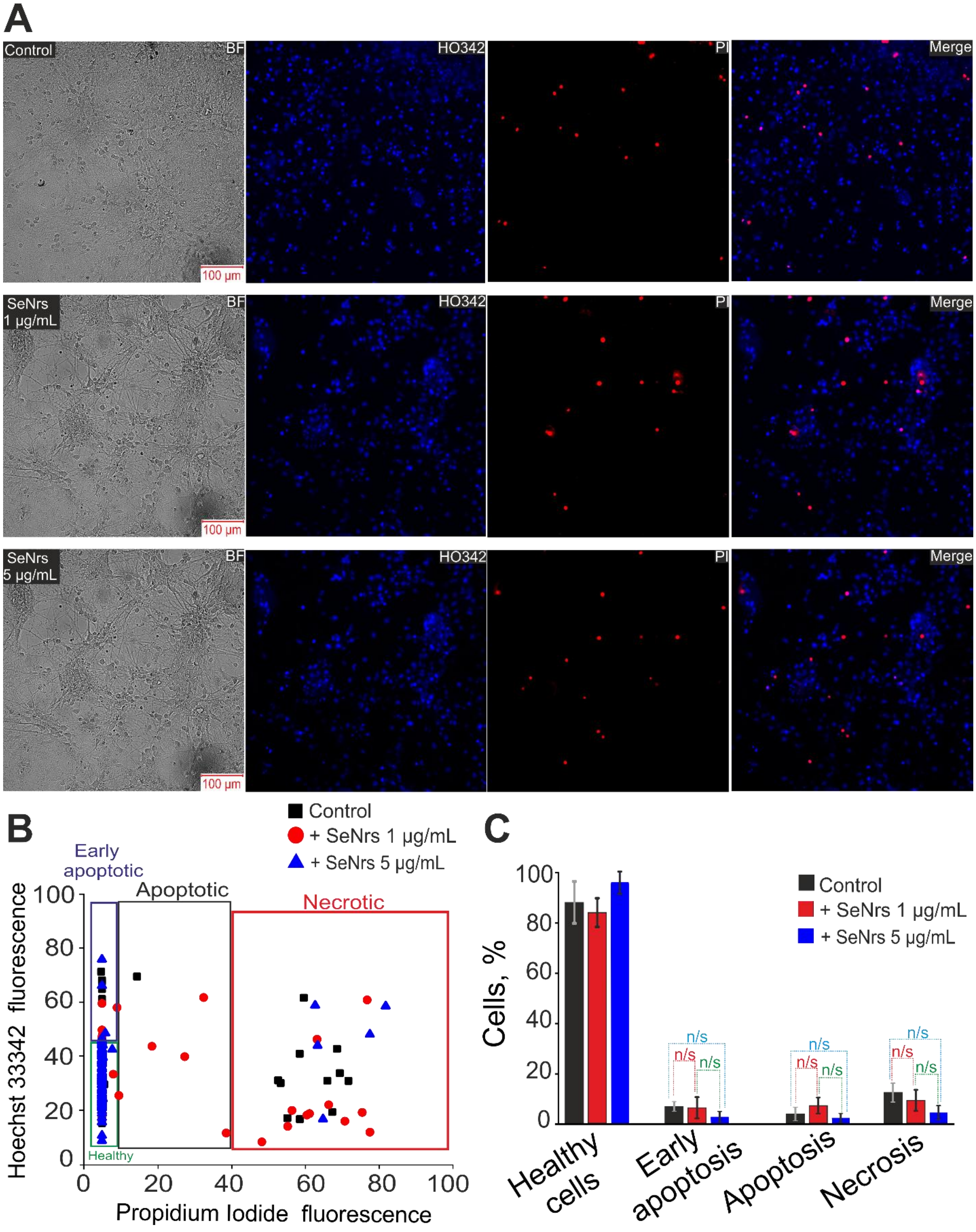

SUPPLEMENTARY, FIGURE S1. The effect of 24-hour preincubation of cortical astrocytes with 1 or 5 µg/mL of selenium nanorods (SeNrs) on the induction of necrosis and apoptosis. (**A**) – Double staining of cells with Hoechst 33342 (HO342), Propidium iodide (PI), merge HO342 with PI and bright-field microscopy (BF). Control – cells without SeNrs. (**B**) – Cytogram demonstrating the viability of cortical astrocytes in Control (without SeNrs) and after 24-h preincubation with 1 or 5 µg/mL SeNrs. X-axis – the intensity of PI fluorescence; Y-axis – the intensity of Hoechst 33342 fluorescence. Cells were stained with the probes 24 hours after the application of SeNrs. (**C**) – Effect

of 24 hours preincubation with 1 or 5 μg/mL SeNrs on the induction of necrosis and apoptosis. N cell cultures = 5; n cover slips with cells for each sample = 5. Preincubation of cells with the SeNrs does not cause reliable cell death (n/s – data not significant, $p > 0.05$).

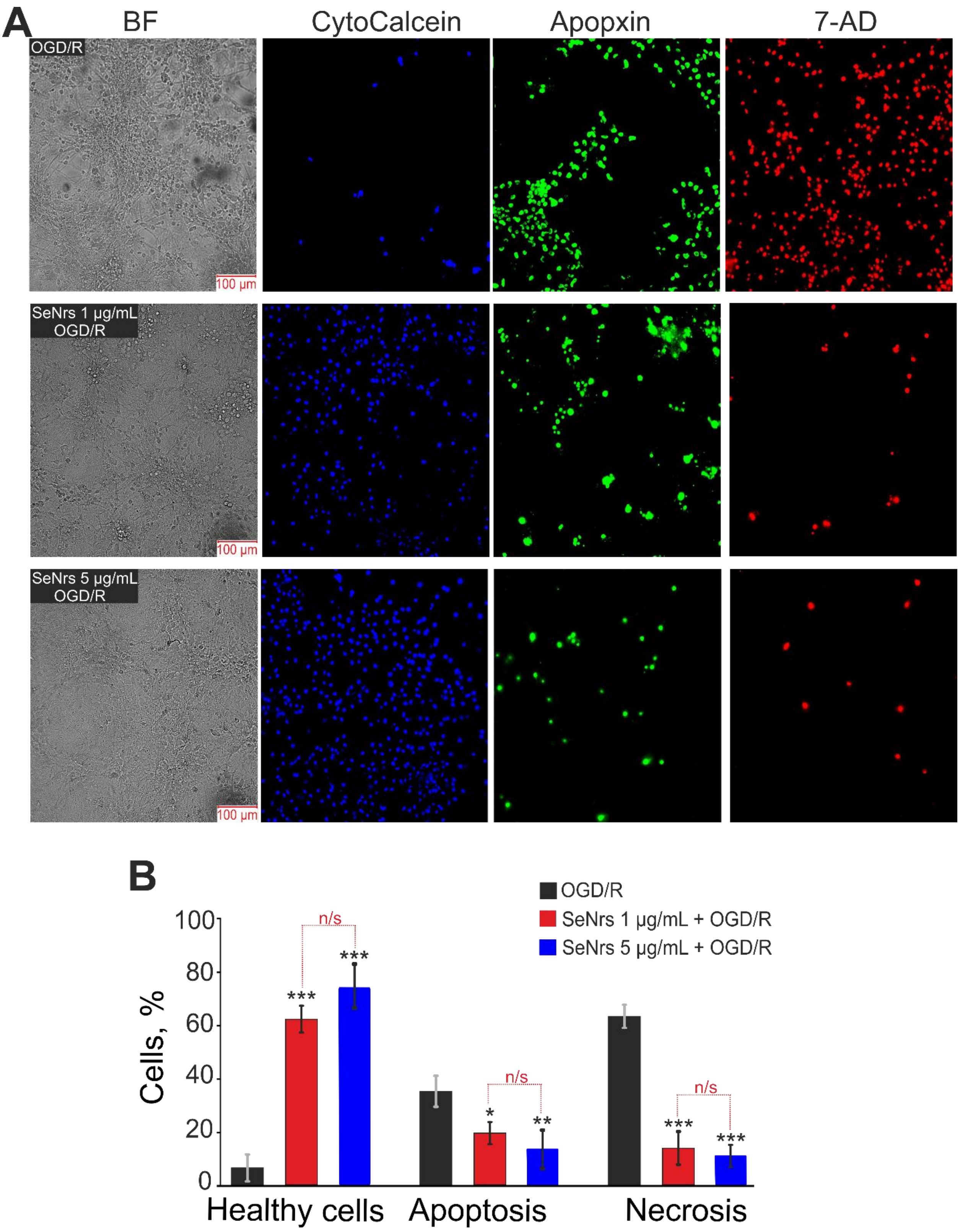

SUPPLEMENTARY, FIGURE S2. Effect of 24-h incubation of cortical astrocytes with 1 μg/mL or 5 μg/mL of SeNrs and OGD/R on the induction of necrosis and apoptosis after 24 h OGD/R. (**A**) – Cell staining using the Apoptosis/Necrosis Detection Kit assay. BF – bright-field microscopy, CytoCalcein – healthy cells indicator, Apopxin – apoptotic cells indicator and 7-AD (7-aminoactinomycin D) – necrotic cells indicator. (**B**) – Healthy cells and cells with apoptosis or necrosis after incubation with 1 μg/mL or 5 μg/mL of SeNrs and OGD/R. Each value is the mean ± SE ($n \geq 3$). Black asterisks represent comparison of experimental groups versus OGD/R-group. Comparison of experimental groups with control: n/s – data not significant ($p > 0.05$), * $p < 0.05$, ** $p < 0.01$, and *** $p < 0.001$. Comparison of experimental groups with each other is indicated in red.
